# Risk Assessment of Precancers and Cancers in Women with Atypical Glandular Cells of Endocervical, Endometrial, and Unknown Origin

**DOI:** 10.7150/jca.111557

**Published:** 2025-07-11

**Authors:** Chen Ding, Qin Liu, Xin Zhou, Qiuqin Zou, Wanrun Lin, Huijuan Zhang, Yudong Wang, Feng Zhou

**Affiliations:** 1 Departments of Pathology, The International Peace Maternal and Child Health Hospital, School of Medicine, Shanghai Jiao Tong University, Shanghai, China, 200030.; 2 Shanghai Key Laboratory of Embryo Original Diseases, Shanghai, China, 200030.; 3 Departments of Pathology, Zhejiang university school of medicine women's hospital, Hangzhou, Zhejiang, China, 310006.; 4 Laboratory of Pathology, National Cancer Institute, National Institutes of Health, Bethesda, MD, USA, 20892.; 5 Department of Oncology, The International Peace Maternal and Child Health Hospital, School of Medicine, Shanghai Jiao Tong University, Shanghai, China, 200030.

**Keywords:** abnormal cervical glandular cytology, hrHPV, age, risk stratification

## Abstract

**Objective:** To assess the risk of precancers [high-grade squamous intraepithelial lesions (HSIL), adenocarcinoma *in situ* (AIS) and atypical endometrial hyperplasia (AEH)] and cancers [squamous cell carcinoma (SCC) and adenocarcinoma (ADC)] in women with atypical glandular cells (AGC) cytology.

**Methods**: A total of 1,028 women diagnosed with abnormal cervical glandular cytology from January 2019 and December 2023 were enrolled. Of these, 670 underwent both HPV genotyping and cervical biopsy.

**Results:** Participants were classified into three groups: AGC-EC (endocervical), AGC-EM (endometrial), and AGC-NOS (unknown origin). AGC-EC was more prevalent than AGC-EM and AGC-NOS among younger women (cutoffs at 40 and 50; P < 0.0001 and < 0.0001) and in the HPV-positive group (*P* = 0.001). In the HPV-positive group, AGC-EC and AGC-NOS lesions were primarily endocervical, with significantly higher incidences of HSIL+, SCC, and AIS+ compared to the HPV-negative group (P = 0.00021, 0.047, < 0.0001 for AGC-EC; P ≤ 0.0001, = 0.004, < 0.0001 for AGC-NOS). However, hrHPV status did not significantly affect the incidence of endometrial and extrauterine lesions. Stratified by age, AGC-NOS's ECA and EUC were more common after age 65 (P = 0.028 and 0.001), and AGC-EM's AEH+ and EMC also increased significantly after 65 (P = 0.001, 0.000401). Moreover, for AGC-EM, older women (≥ 50) had significantly higher rates of AEH+ and EMC compared to younger groups (*P* < 0.05).

**Conclusion**: Distinct cytological categories of AGC exhibit differential age and HPV-related risk profiles. AGC-EC and AGC-NOS in HPV-positive women indicate a higher risk of cervical neoplasia, highlighting the importance of HPV testing in triaging these cases. In contrast, AGC-EM is predominantly linked to endometrial pathologies in older women, especially those aged ≥ 50. These findings underscore the necessity of age- and subtype-specific evaluation strategies to optimize early detection of glandular and extrauterine malignancies in AGC patients.

## Introduction

Cervical cancer remains a significant global health concern, particularly in developing regions 1. With extensive cervical cancer screening, its incidence and mortality have declined 2, 3, yet the incidence of cervical adenocarcinoma has increased 4, 5. While the risk of cervical cancer associated with squamous cell abnormalities on cytology specimens is well-established 6, 7, the risk tied to glandular abnormalities is less clearly defined.

Atypical glandular cells (AGC) are relatively uncommon in cervical cytology tests, with detection rates generally below 1% 8. Diagnoses in subsequential biopsy range from benign findings to precancerous lesions and cervical cancer, as well as endometrial cancer and other genital malignancies 9-11. In the 2014 Bethesda Nomenclature System for Cervical Cytology, glandular cell abnormalities are further subtyped by origin and malignancy risk: (1) AGC-not otherwise defined (AGC-NOS), (2) AGC-endocervical cells (AGC-EC), (3) AGC-endometrial cells (AGC-EM), and (4) AGC-favor neoplasia (AGC-FN) 12. This origin-based classification enables precise diagnosis, supports treatment decisions 13, facilitates disease risk evaluation, and guides personalized follow-up 14. It also offers insights into disease pathogenesis, thus aiding improvements in cervical cancer screening and diagnostic systems 15.

The risk of cervical Precancers and Cancers in AGC varies by location. Although some studies have examined the influence of HPV infection and age on AGC-related risk 16, 17, site-based comparisons remain limited. Assessing AGC risk by origin is essential for optimizing management. This study investigates the risk of cervical precancers and cancers in AGC subgroups categorized by HPV status and age.

## Materials and Methods

### Samples collection

**Figure 1:**
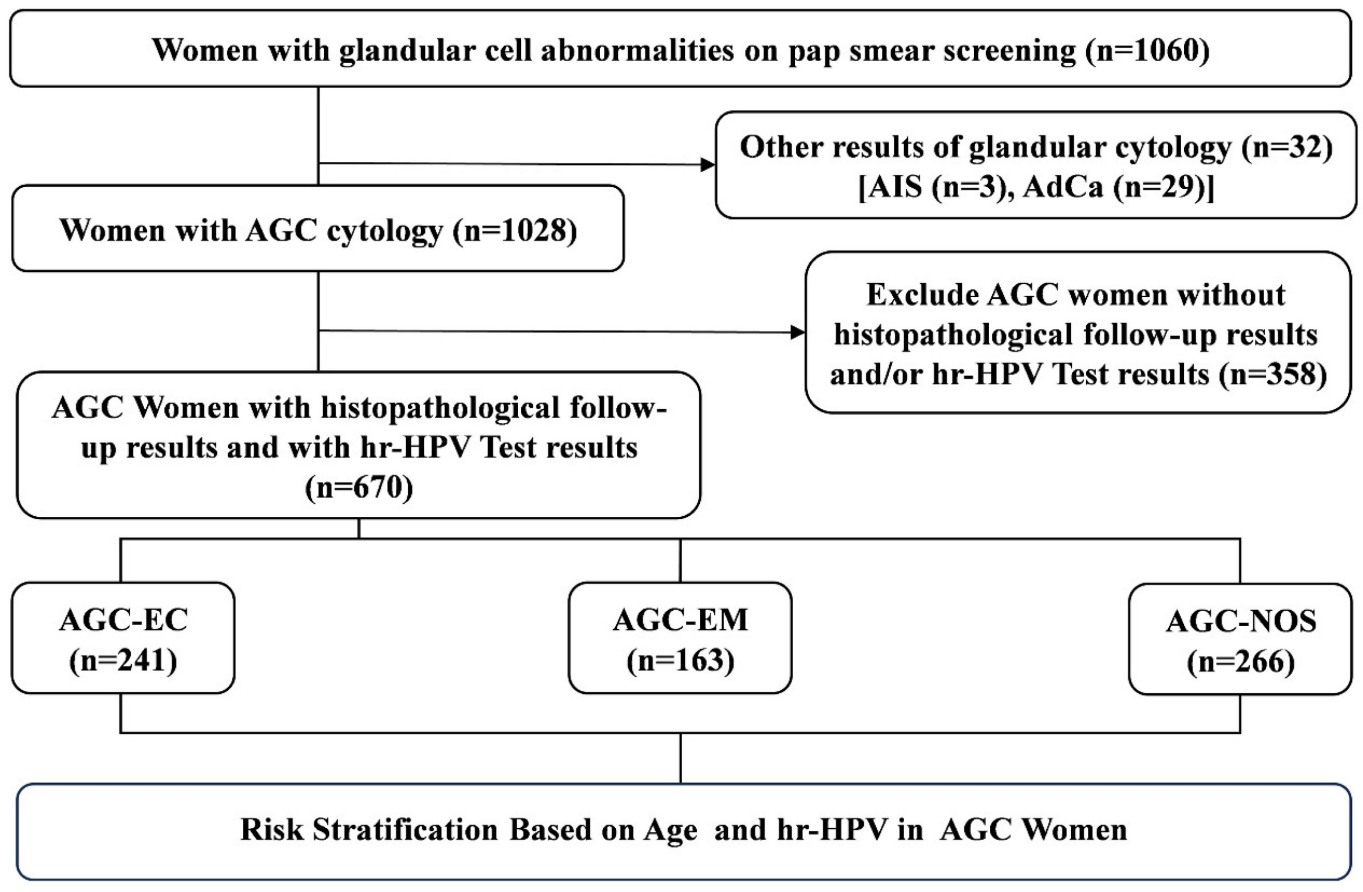
** Flow chart of selection criteria of participants.** AdCa, adenocarcinoma; AGC-NOS, AGC -unknown origin; AGC-EC, AGC-endocervical cells; AGC-EM, AGC-endometrial cells; AIS, adenocarcinoma *in situ*.

We performed a retrospective review of our pathology database, identifying all patients diagnosed with AGC between January 2019 and December 2023. Inclusion Criteria: Females with a history of sexual activity who were non-pregnant; all subjects with a clear diagnosis of cervical biopsy pathology; subjects underwent Liquid-Based Cytology Test (LCT) testing, with results indicating AGC; subjects with no autoimmune disorders. Exclusion Criteria: subjects with unreliable follow-up; subjects with history of hysterectomy or malignant tumors; subjects with history of other malignancies or pelvic radiotherapy/chemotherapy. We retrieved data from the electronic medical records system, including patient age at the time of AGC diagnosis, available HPV testing results, and follow-up histopathological outcomes. As shown in Figure 1, a total of 1,028 women with abnormal cervical glandular cytology were identified, of them 799 underwent simultaneous HPV genotyping and 769 had available histopathological results. After excluding cases without HPV testing or missing histopathological data, 670 patients were ultimately included in the study.

### Liquid-based cytology and hrHPV mRNA testing

LCT testing were performed per the manufacturer's protocol. All cytology slides were evaluated by cytopathologists in our hospital, following the 2014 Bethesda system 12. Fourteen hrHPV genotypes (16, 18, 31, 33, 35, 39, 45, 51, 52, 56, 58, 59, 66, and 68) were tested. Positive hrHPV samples were categorized into four types: HPV16, HPV18, HPV16/18 dual-positive, and other hrHPV types.

### Subsequential histopathological diagnoses in patients

**Figure 2:**
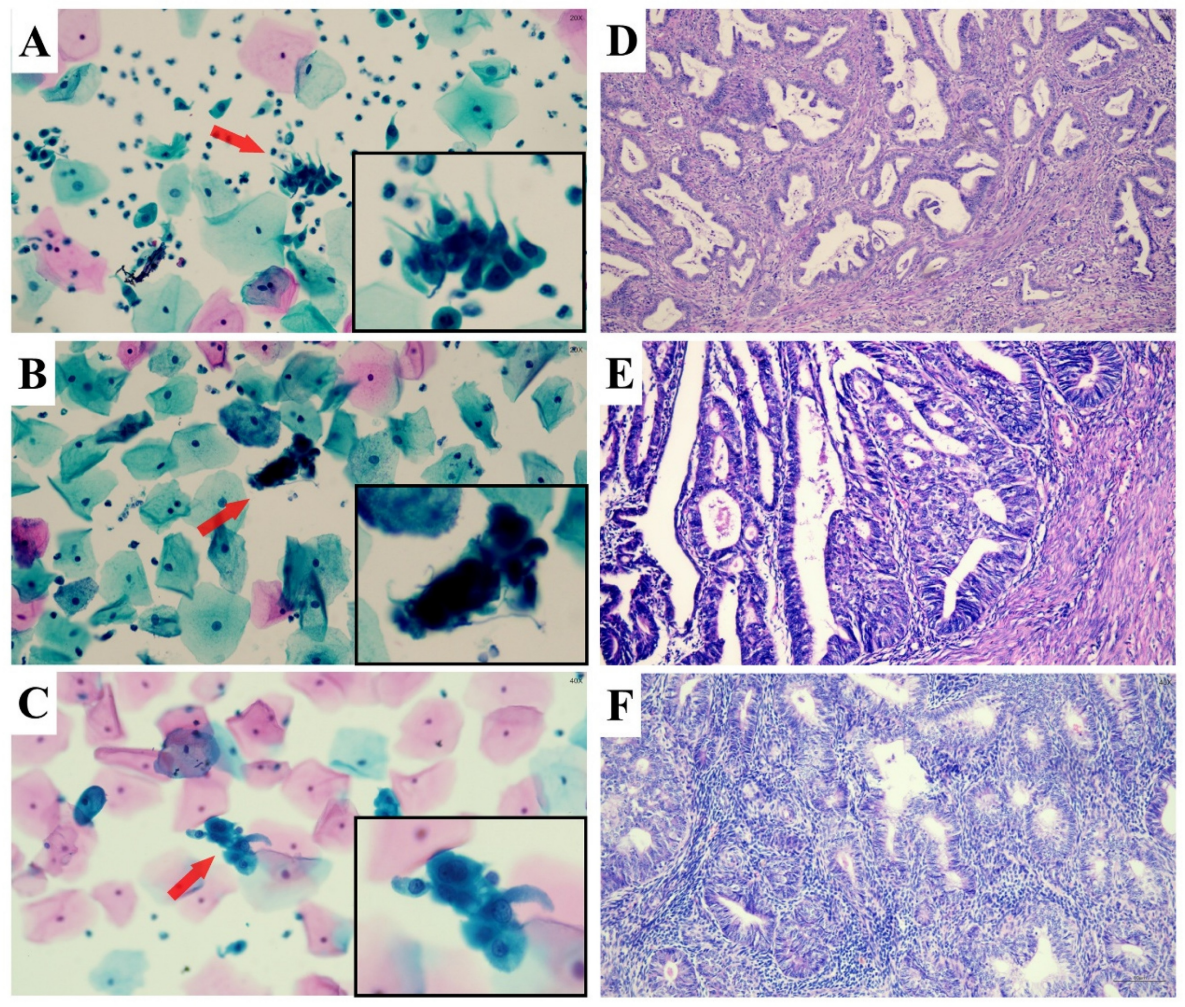
** The representative images of abnormal glandular cells (AGC) confirmed by the follow-up histologic examinations.** AGC-endocervical cells **(A)**: In the area indicated by arrows and the black box, abnormal cells arranged in sheets display hyperchromatic, crowded, and enlarged nuclei with irregular nuclear membranes and an increased nuclear-to-cytoplasmic ratio; their cell borders appear feathery. AGC-endometrial cells **(B)**: Abnormal cells forming three-dimensional clusters in the marked area exhibit nuclear atypia. AGC-not otherwise defined **(C)**: A cluster of crowded cells with indistinct borders in the marked area shows nuclear atypia, but the cellular origin remains indeterminate. Subsequent histopathological diagnoses were endocervical adenocarcinoma **(D)**, endometrioid adenocarcinoma **(E)**, and endometrioid adenocarcinoma **(F)**, respectively.

Histopathological samples were obtained via various procedures, including colposcopic endocervical curettage, cervical biopsy, endometrial sampling, loop electrosurgical excision procedure (LEEP) or conization, and total hysterectomy. We categorized the histopathologic results into: (1) Benign lesion (including benign, low-grade squamous intraepithelial lesion (LSIL) and endometrial hyperplasia (EH)) , (2) High-grade squamous intraepithelial lesion (HSIL+), defined as HSIL or squamous cell carcinoma (SCC); (3) High-grade cervical glandular abnormalities (AIS+), encompassing adenocarcinoma *in situ* (AIS) and invasive endocervical adenocarcinoma (ECA); (4) High-grade endometrial glandular abnormalities (AEH+), referring to atypical endometrial hyperplasia (AEH) and endometrial carcinoma (EMC). (5) Extrauterine adenocarcinoma (EUA). In cases with multiple tissue samples, the most severe diagnosis was recorded. Representative images of abnormal glandular cells confirmed by the follow-up histologic examinations were shown in Figure 2.

### Statistical analysis

Pearson's χ2 or Fisher's exact test was conducted using SPSS (version 23.0, IBM) to compare the distributions of HSIL+ and AIS+ across different age groups. The Kruskal-Wallis test was used to compare the distribution of histological outcomes among the AGC population with differing HPV infection phenotypes. A *p* < 0.05 was deemed statistically significant.

## Results

### Demographic characteristics of patients with AGC

A total of 670 cases with cytological AGC positivity were analyzed. Among them, 241 cases (36.0%) were classified as AGC-EC, 163 cases (24.32%) were classified as AGC-EM, and 266 cases (39.70%) as AGC-NOS. The mean ages of these groups were 42.76, 47.91, and 45.87 years, respectively. Although a statistically significant difference (*P* < 0. 0001) was observed between the groups (*P* < 0. 0001), all groups were within the 40-50-year range. Further analysis of age's impact on AGC classification revealed significant differences when using 40 or 50 years as cutoff points (*P* < .0001). Of the cases, 670 patients both underwent hrHPV testing with an overall prevalence of 19.40% (130/670). In the HPV-positive group, AGC-EC incidence also exceeded that of the other two (26.14% vs 11.66%, 18.05%, P = 0.001).

**Figure 3:**
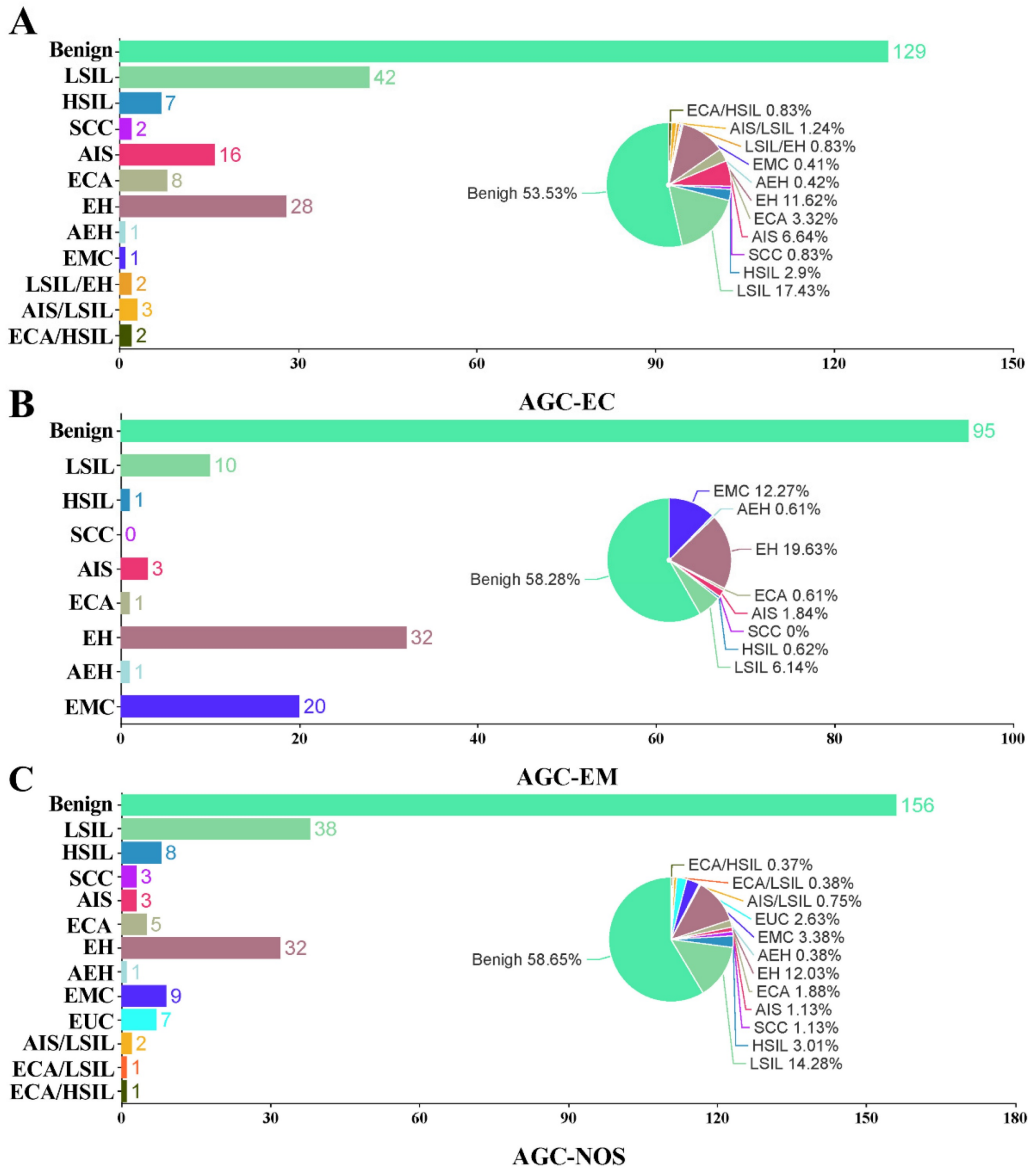
** Bar and pie charts show the proportion and case number of histological results in women with AGC cytology.** AGC-EC women **(A)**, AGC-EM women **(B)**, and AGC-NOS women **(C)**. AGC, atypical glandular cells; AGC-NOS, AGC -unknown origin; AGC-EC, AGC-endocervical cells; AGC-EM; AGC-endometrial cells; AEH, atypical endometrial hyperplasia; AIS, adenocarcinoma *in situ*; ECA, endocervical adenocarcinoma; EH, endometrial hyperplasia; EMC, endometrial carcinoma; EUA, extrauterine adenocarcinoma; HSIL, high-grade squamous intraepithelial lesion; LSIL, low-grade squamous intraepithelial lesion; SCC, squamous cell carcinoma; NS, not significant.

All the patients had subsequential histopathological diagnoses. In the AGC-EC group (n = 241), 13 (5.39%) had malignant results, 27 (11.2%) had precancerous findings, and 201 (83.40%) were benign. In the AGC-EM group (n = 163), 21 (12.88 %) were malignant, 5 (3.07%) had precancerous lesions, and 137 (84.05%) were benign. In the AGC-NOS group (n = 266), 26 (9.77%) were malignant, 14 (5.26%) were precancerous, and 226 (84.96%) were benign. Malignancy rates were significantly higher in the AGC-EM group compared to the other two (P = 0.014). Detailed analysis is included in Table 1 and Figure 3.

### HPV-stratified prevalence of precancers and cancers by histological diagnosis in women with AGC

A total of 266 patients in the AGC-NOS group underwent hrHPV testing, with 48 positive and 218 negative results. Among the 48 hrHPV-positive patients, 12 were diagnosed with HSIL+ squamous lesions, 7 with AIS+ glandular lesions, and 2 with AEH+. In the 218 hrHPV-negative patients, no HSIL+ lesions were found, while 5 had AIS+, 8 had AEH+, and 7 had EUC. Patients with HPV-positive AGC-NOS, histopathologically diagnosed in endocervical lesions, had higher incidences of HSIL+, SCC, and AIS+ than that of HPV-negative AGC-NOS (P = 1.0×10⁻¹⁴, 0.004, 7.8×10⁻⁵).

We also analyzed the hrHPV results in the AGC-EC group, which included 241 patients followed up for histological results. Among the 63 hrHPV-positive patients, 8 (12.70%) were diagnosed with HSIL+, and 16 (25.40%) with AIS+. Of the 178 hrHPV-negative patients, 3 (1.69%) were diagnosed with HSIL+, and 13 (7.30%) with AIS+, while 1 (0.56%) was diagnosed with AEH+. The analysis revealed a significantly higher incidence of HSIL+, SCC, and AIS+ in the hrHPV-positive group compared to the negative group (P = 0.00021, 0.047, < 0.0001). In the AGC-EM group, no significant difference was observed between the HPV-positive and HPV-negative groups. Detailed analysis is included in Table 2.

### Age-stratified prevalence of precancers and cancers by histological diagnosis in Women with AGC

We divided the subjects into age groups (< 25, 25-39, 40-65, and > 65 years) to examine differences in diagnoses. In the AGC-NOS group, the > 65 age group had a significantly higher ECA rate for endocervical lesions (P = 0.028), and a higher EUC for extrauterine lesions (*P* = 0.001). As for AGC-EM group, the prevalence of AEH+ and EMC is significantly higher in the > 65 age group than other age groups for endometrial lesions (P = 0.001, P = 0.000401). Detailed analysis is included in Table 3.

To further explore the impact of age on the progression to precancers and cancers, we selected 25, 40, and 50 years as the cutoff points for analysis. In both the AGC-NOS and AGC-EC groups, there is no significant difference in the rates of precancers and cancers when diagnosed as endocervical lesions, endometrial lesions or extrauterine lesions. However, in AGC-EM group when histopathologically diagnosed as endometrial lesions or extrauterine lesions, the incidence of AEH+ and EMC is significantly higher in the older age groups (≥ 50 years) compared to the younger age groups (P < 0.0001 and < 0.0001). Detailed analysis results are shown in Table 4.

## Discussion

Despite the decreasing incidence of cervical squamous lesions, cervical adenocarcinoma has risen from 5% to 20-30% 18. This shift is greatly attributed to the lower sensitivity of the Pap smear for glandular lesions, which was primarily designed to detect squamous lesions 19. Adenocarcinomas, which often develop in the upper cervix, pose sampling challenges, and benign glandular cells can mimic atypical or malignant cells. Studies indicate some AGC cases also involve squamous abnormalities 20, highlighting a need for more refined screening. Although HPV testing effectively detects many cervical cancers, about 15% of adenocarcinomas are HPV-negative 21. Therefore, identifying risk factors and refining clinical management for AGC patients is vital to enhancing cervical cancer screening and prevention.

This study examined the risk of precancerous lesions and cancer in women with AGC cytology, stratified by hrHPV status and age, thus offering multidimensional insights with potential clinical significance. Notably, the overall HPV positivity rate for all AGC types was 19.4% (130/670), which is lower than rates reported in European populations 22, 23. In contrast, another study from China using Cobas 4800 HPV testing found a higher hrHPV infection rate of 41.1% (51/124) in AGC women 24. Such discrepancies may be attributable to geographic, population-based, or testing platform differences. Nevertheless, in the HPV-positive group, AGC-EC incidence notably surpassed that of AGC-EM and AGC-NOS, suggesting that endocervical cytological endocervical cytological manifestations are increased in patients with HPV infection.

To investigate how HPV status might influence progression in different AGC subtypes, we classified lesions as endocervical, endometrial, or extrauterine based on histopathology. Overall, 15.8% (106/670) of AGC cases were diagnosed with precancer or cancer, mirroring a study from Thailand 25, but lower than the 21.9% rate reported in the United States 26. Interestingly, although HPV infection showed a strong correlation with endocervical lesions, it did not significantly affect precancer or cancer rates in endometrial or extrauterine adenocarcinomas. This finding implies that endocervical adenocarcinomas and AIS, which are primarily HPV-associated, would be increased in HPV-positive patients whereas cancers of the endometrium and other sites which are not associated with HPV remain unchanged.

Regarding the distribution of AGC subtypes across different age groups, we found that the average age of AGC-EC was 42.76 years, which is younger than AGC-NOS and AGC-EM, consistent with prior studies 27. Cervical tissue in younger women tends to be more active and susceptible to HPV infection, potentially contributing to the development of AGC-EC. Concerning age stratification, significant differences emerged in the incidence of precancers and cancers in AGC. Among individuals aged 65 and older, AIS+ and ECA were more frequent in AGC-NOS for endocervical lesions, potentially reflecting immune system decline and hormonal changes. Similar findings have been reported for endometrial lesions in women with AGC aged 50 and older 28. Additionally, within AGC-EM patients in this age range, higher incidences of AEH+ and EMC suggest that hormone-dependent processes may influence endometrial lesion development in older women. In extrauterine lesions, AGC-NOS and AGC-EC both showed increased EUC prevalence in the 65+ group. Further, when 50 years were used as cutoff points, AEH+ and EMC rose significantly in older age groups (> 50) compared to younger groups. Overall, these results indicate distinct age-related distribution patterns for high-grade AGC lesions at various anatomical sites.

This study presents several key innovations in assessing the risk of precancerous and malignant lesions among women with AGC. First, AGC cases were further stratified into AGC-EC, AGC-EM, and AGC-NOS, allowing a more systematic and anatomically relevant classification. This subdivision enhances clinical interpretation by linking cytologic findings to their likely sites of origin. In accordance with the Bethesda System recommendations 12, specifying the presumed origin of AGC in cytological reports may guide more targeted follow-up strategies—for instance, prioritizing endometrial evaluation in AGC-EM cases, while recommending cervical biopsy and imaging for AGC-EC and AGC-NOS cases. Second, by integrating hr-HPV status and patient age into the risk assessment framework, this study highlights the heterogeneous distribution of cervical, endometrial, and extrauterine lesions across different subpopulations. Risk-stratified management should therefore be guided by both HPV status and age. For example, HPV-positive AGC patients may benefit from prompt colposcopic evaluation and cervical biopsy, whereas older women diagnosed with AGC-EM or AGC-NOS warrant careful assessment for endometrial or extrauterine malignancies, potentially including transvaginal ultrasound, MRI, and hysteroscopy. Specifically, our findings revealed a significantly higher prevalence of AGC-EC in younger, HPV-positive women, with these cases showing an increased risk of high-grade cervical lesions or invasive cancer. Therefore, HPV-positive patients with AGC-EC or AGC-NOS should undergo intensified surveillance and more comprehensive cervical assessment, such as multi-site biopsies or diagnostic excisional procedures. In contrast, AGC-EM (≥ 50) or AGC-NOS (≥ 65) in older women were more frequently associated with endometrial carcinoma or extrauterine tumors, underscoring the need to incorporate routine endometrial evaluation—via hysteroscopy and endometrial sampling—into their clinical management to prevent missed diagnoses. Despite these insights, our study has some limitations. The single-center design may limit generalizability, and larger multicenter research would confirm the broader applicability of these findings. In addition, we did not analyze the correlation between specific HPV genotypes and different AGC subtypes. Future research should examine genotype-specific influences on precancer and cancer progression in AGC.

Inclusion, AGC subtypes show distinct age and HPV-related risk patterns, and these findings underscore the need for tailored management strategies. Younger, HPV-positive women with AGC-EC may require closer surveillance of cervical lesions, whereas older women with AGC-EM (≥ 50) or AGC-NOS (≥ 65) may benefit from thorough assessments of endometrial and extrauterine regions.

## Figures and Tables

**Table 1:** Demographic Characteristics of Patients with Atypical Glandular Cells

Characteristic	AGC-EC	AGC-EM	AGC-NOS	*P*-value
Age, mean, y	42.76 ± 9.60	47.91 ± 10.38	45.87 ± 10.71	**< 0.0001**
Age, y				**0.030**
< 25	2	0	2	
25-39	94	31	72	
40-65	137	119	175	
> 65	8	13	17	
Age, y				0.683
< 25 years	2	0	2	
≥ 25 years	239	163	264	
Age, y				**< 0.0001**
< 40 years	96	31	74	
≥ 40 years	145	132	192	
Age, y				**< 0.0001**
< 50 years	193	102	179	
≥ 50 years	48	61	87	
HPV status, n/N (%)				**0.001**
Negative	178	144	218	
Positive	63	19	48	
HPV type, n/N (%)				0.560
16+	13	4	11	
18+	13	1	6	
Other hrHPV+	35	14	31	
16+,18+	2	0	0	
Histopathological Outcome				**0.014**
Negative/Benign	201	137	226	
Premalignancy	27	5	14	
Malignancy	13	21	26	

AGC, atypical glandular cells; AGC-NOS, AGC -unknown origin; AGC-EC, AGC-endocervical cells; AGC-EM; AGC-endometrial cells; hrHPV, high-risk human papillomavirus.

**Table 2:** HPV-stratified prevalence of precancers and cancers by histological diagnosis in women with AGC

Group	hrHPV	Total	Endocervical lesions								Endometrial lesions				Extrauterine lesions	
			HSIL+	p	SCC	p	AIS+	p	ECA	p	AEH+	p	EMC	p	EUC	p
**AGC-EC**	negative	178	3	**0.00021**	0	**0.047**	13	**< 0.0001**	5	0.119	1	0.364	0	0.202	0	NA
	positive	63	8		2		16		5		1		1		0	
	Total	241	11		2		29		10		2		1		0	
**AGC-EM**	negative	144	0	0.130	0	NA	4	0.63	1	1.0	20	0.511	19	0.556	0	NA
	positive	19	1		0		0		0		1		1		0	
	Total	163	1		0		4		1		21		20		0	
**AGC-NOS**	negative	218	0	**< 0.0001**	0	**0.004**	5	**< 0.0001**	3	**0.0075**	8	0.823	7	0.715	7	1.000
	positive	48	12		3		7		4		2		2		0	
	Total	266	12		3		12		7		10		9		7	

AGC, atypical glandular cells; AGC-NOS, AGC -unknown origin; AGC-EC, AGC-endocervical cells; AGC-EM; AGC-endometrial cells; AEH, atypical endometrial hyperplasia; AIS, adenocarcinoma *in situ*; ECA, endocervical adenocarcinoma; EMC, endometrial carcinoma; EUA, extrauterine adenocarcinoma; HSIL, high-grade squamous intraepithelial lesion; SCC, squamous cell carcinoma; NS, not significant; hrHPV, high-risk human papillomavirus.

**Table 3:** Age-stratified prevalence of precancers and cancers by histological diagnosis in women with AGC

Group	Age	Total	Endocervical lesions								Endometrial lesions				Extrauterine lesions	
			HSIL+	p	SCC	p	AIS+	p	ECA	p	AEH+	p	EMC	p	EUC	p
**AGC-EC**	< 25	2	0	0.727	0	0.983	0	0.558	0	0.445	0	0.694	0	0.868	0	1.000
	25-39	94	4		1		13		2		0		0		0	
	40-65	137	6		1		14		7		2		1		0	
	> 65	8	1		0		2		1		0		0		0	
	Total	241	11		2		29		10		2		1		0	
**AGC-EM**	< 25	0	0	0.838	0	1.000	0	0.891	0	0.837	0	**0.001**	0	**0.000401**	0	1.000
	25-39	31	0		0		1		0		1		1		0	
	40-65	119	1		0		3		1		14		13		0	
	> 65	13	0		0		0		0		6		6		0	
	Total	163	1		0		4		1		21		20		0	
**AGC-NOS**	< 25	2	0	0.523	0	0.705	0	0.068	0	**0.028**	0	0.214	0	0.170	1	**0.001**
	25-39	72	1		0		6		3		3		2		1	
	40-65	175	10		3		4		2		5		5		4	
	> 65	17	1		0		2		2		2		2		1	
	Total	266	12		3		12		7		10		9		7	

AGC, atypical glandular cells; AGC-NOS, AGC -unknown origin; AGC-EC, AGC-endocervical cells; AGC-EM; AGC-endometrial cells; AEH, atypical endometrial hyperplasia; AIS, adenocarcinoma *in situ*; ECA, endocervical adenocarcinoma; EMC, endometrial carcinoma; EUA, extrauterine adenocarcinoma; HSIL, high-grade squamous intraepithelial lesion; SCC, squamous cell carcinoma; NS, not significant.

**Table 4:** Immediate risk of precancer and cancer between older and younger group with AGC cytology

Group	Age	Total	Endocervical lesions								Endometrial lesions				Extrauterine lesions	
			HSIL+	*p*	SCC	*p*	AIS+	*p*	ECA	*p*	AEH+	*p*	EMC	*p*	EUC	*p*
**AGC-EC**	25-year cutoff			1.000		1.000		1.000		1.000		1.000		1.000	0	NA
	≥ 25 years	239	11		2		29		10		2		1		0	
	40-year cutoff			1.000		1.000		0.570		0.320		0.522		1.000	0	NA
	≥ 40 years	145	7		1		16		8		2		1		0	
	50-year cutoff			0.870		1.000		0.690		0.220		1.000		1.000	0	NA
	≥ 50 years	48	3		0		5		4		0		0		0	
**AGC-EM**	25-year cutoff			NA		NA		NA		NA		NA		NA	0	NA
	≥ 25 years	163	1		0		4		1		21		20		0	
	40-year cutoff			1.000		NA		1.000		1.000		0.140		0.085	0	NA
	≥ 40 years	132	1		0		3		1		20		19		0	
	50-year cutoff			0.297		NA		0.732		0.298		**< 0.0001**		**< 0.0001**	0	NA
	≥ 50 years	61	1		0		2		1		18		17		0	
**AGC-NOS**	25-year cutoff			1.000		1.000		1.000		1.000		1.000		1.000	1	0.059
	≥ 25 years	264	12		3		12		7		10		9		6	
	40-year cutoff			0.260		0.567		0.180		0.670		1.000		1.000	2	1.000
	≥ 40 years	192	11		3		6		4		7		7		5	
	50-year cutoff			0.670		0.236		0.670		0.300		0.830		0.640	4	0.820
	≥ 50 years	87	5		2		5		4		4		4		3	

AGC, atypical glandular cells; AGC-NOS, AGC-NOS, AGC -unknown origin; AGC-EC, AGC-endocervical cells; AGC-EM; AGC-endometrial cells; AEH, atypical endometrial hyperplasia; AIS, adenocarcinoma in situ; ECA, endocervical adenocarcinoma; EMC, endometrial carcinoma; EUA, extrauterine adenocarcinoma; HSIL, high-grade squamous intraepithelial lesion; SCC, squamous cell carcinoma; NS, not significant.
